# Estimation of the Covariance Matrix in Hierarchical Bayesian Spatio-Temporal Modeling via Dimension Expansion

**DOI:** 10.3390/e24040492

**Published:** 2022-03-31

**Authors:** Bin Sun, Yuehua Wu

**Affiliations:** Department of Mathematics and Statistics, York University, Toronto, ON M3J 1P3, Canada; bsun1010@gmail.com

**Keywords:** entropy, environmental network design, dimension expansion, hierarchical Bayesian spatio-temporal modeling, nonstationary field, semivariogram

## Abstract

Ozone concentrations are key indicators of air quality. Modeling ozone concentrations is challenging because they change both spatially and temporally with complicated structures. Missing data bring even more difficulties. One of our interests in this paper is to model ozone concentrations in a region in the presence of missing data. We propose a method without any assumptions on the correlation structure to estimate the covariance matrix through a dimension expansion method for modeling the semivariograms in nonstationary fields based on the estimations from the hierarchical Bayesian spatio-temporal modeling technique (Le and Zidek). Further, we apply an entropy criterion (Jin et al.) based on a predictive model to decide if new stations need to be added. This entropy criterion helps to solve the environmental network design problem. For demonstration, we apply the method to the ozone concentrations at 25 stations in the Pittsburgh region studied. The comparison of the proposed method and the one is provided through leave-one-out cross-validation, which shows that the proposed method is more general and applicable.

## 1. Introduction

Ozone concentrations are the daily maximum 8 h moving averages of hourly ozone concentration data recorded in micrograms per cubic meter, μg/m${}^{3}$, which are key indicators of air quality. Monitoring the changes both spatially and temporally is very important for the assessment of air quality change, which has a great impact on our environment, society and economy. However, modeling the ozone concentrations is not an easy task since the ozone concentrations vary over space and time with complicated spatial structures, temporal structures and spatio-temporal interactions. Furthermore, the presence of missing data brings even more difficulties. As commented in [1], although we cannot escape the “curse of dimensionality”, we can take advantage of recent developments in computing speed and numerical advances (e.g., Markov chain Monte Carlo) that allow us to implement Bayesian spatio-temporal dynamical models in a hierarchical framework. Such a framework provides simple strategies for incorporating complicated spatio-temporal interactions at different stages of the models’ hierarchy, and the models are feasible to be implemented for high-dimensional data. Two popular hierarchical Bayesian spatio-temporal models can be found in [1,2], among others. The latter one was used in [3].

Ref. [3] studied the ozone concentrations within $-79^{\circ }$ to $-81.5^{\circ }$ longitude and $39.5^{\circ }$ to $41.5^{\circ }$ latitude around the Pittsburgh region ($-79.23^{\circ }$, $43.39^{\circ }$), in which all of the monitoring stations have missing data. That paper dealt with the missing problems in two steps. First, it filled in some of the missing measurements by using linear models so that the pattern of missing data became monotone (the monotone missing is also referred to as the staircase pattern). Second, it applied *h*ierarchical *B*ayesian *s*patio-*t*emporal (HBST) modeling proposed in [2] on this staircase of missing data to estimate the hyperparameters of the spatial-temporal model. Based on the estimated hyperparemeters, it estimated the spatial correlation function for the monitoring stations. Then, it estimated the covariance matrix for all of the stations and derived the predictive distribution for the ungauged sites.

Generalized linear models can be used to accommodate non-Gaussian geostatistical data (e.g., see [4]). Ref. [3] selected the generalized linear model with the quasi-Poisson family as an appropriated spatial correlation function by examining the pattern of spatial correlations obtained via the hierarchical model in the plot. However, their link function is not appropriate if there are negative correlations. This is a strong restriction because negative correlations are common for the ozone concentrations and other spatial-temporal data. Moreover, choosing a model by examining the plots derived in terms of the observed data set is not rigorous enough and may only be suitable just for a particular data set.

In this paper, we propose a method to estimate the covariance matrix through a dimension expansion method for modeling the semivariograms in nonstationary fields based on the estimations from hierarchical Bayesian spatio-temporal modeling. For demonstration, we apply the proposed method on the same data as in Jin et al. [3]. Without any assumption on the correlation structure, the proposed method is more general than the method in [3] such that it is applicable to other spatio-temporal data sets. Using the covariance matrix estimated by the proposed method on the entropy criterion in the environmental network design problem, our study provides interesting findings, and the locations of the selected ungauged stations are more reasonable. We provide comparison of these two methods through leave-one-out cross-validation, which shows that the proposed method provides improved results.

The paper is arranged as follows. In Section 2, we briefly introduce hierarchical Bayesian spatio-temporal modeling. In Section 3, we describe the ozone concentrations in the Pittsburgh region and apply the hierarchical Bayesian spatio-temporal modeling techniques for filling in missing measurements following [3]. In Section 4, we model the ozone concentrations in the Pittsburgh region. We first introduce the method for estimating the covariance matrix through a dimension expansion method for modeling the semivariograms in nonstationary fields, and we then give spatial predictive distributions on the ungauged sites using the covariance matrix estimated by the proposed method. In Section 5, we present the results of the entropy of the predictive distributions and an optimality criterion for extending an environmental network. In Section 6, we provide the model evaluation through leave-one-out cross-validation. We conclude this paper with a conclusion in Section 7.

Throughout the rest of the paper, the $L_{1}$-norm of a vector c is denoted by ${∥c∥}_{1}$, a p×p identity matrix is denoted by $I_{p}$, the transpose of a matrix *A* is denoted by $A^{⊤}$ and the trace of a square matrix *B* is denoted by tr(B). In addition, ‘⊗’ represents the Kronecker product, $N_{k\times \ell }\left(\cdot ,\cdot \right)$ refers to a matrix Gaussian distribution, $t_{k\times \ell }\left(\cdot ,\cdot \right)$ denotes a matric-*t* distribution, IW(·,·) stands for the inverted Wishart distribution (see (a) of the appendix for definitions of these distributions) and GIW(·,·) denotes the generalized inverted Wishart distribution.

## 2. Hierarchical Bayesian Spatio-Temporal Modeling

We briefly describe HBST modeling in this section, which is the same as that given in [3] excluding Step 3 in the HBST modeling procedure. It is noted that this modeling is a special case of the HBST modeling presented in Chapter 10 of Le and Zidek (2006) excluding Step 3 in the HBST modeling procedure.

Define the following notations:

*d* = number of different type stations (e.g., agricultural, residential, commercial and industrial);

*n* = number of time points (e.g., number of days);

*u* = number of locations with no monitors (i.e., ungauged sites);

*g* = number of locations with monitors (i.e., gauged sites).

The stations are organized into *k* blocks where the $g_{j}$ (j=1,2,⋯,k) sites in the *j*th block have the same number of timepoints $m_{j}$ at which no measurements are taken. These blocks are numbered so that the measurements correspond to a monotone data pattern or a staircase structure, that is,

$$m_{1}>m_{2}>\cdots >m_{k}\geq 0.$$

The response variables are written as

$$Y=[Y^{\left[u\right]},Y^{\left[g\right]}].$$

Here, $Y^{\left[u\right]}$ of dimension n×u denotes the unobserved responses at ungauged sites while $Y^{\left[g\right]}$ of dimension n×g is given by

$$Y^{\left[g\right]}=\left[Y^{\left[g_{1}\right]},\cdots ,Y^{\left[g_{k}\right]}\right]=\left[\left(\begin{array}{c} Y^{\left[g_{1}^{m}\right]}\\ Y^{\left[g_{1}^{o}\right]} \end{array}\right),\cdots ,\left(\begin{array}{c} Y^{\left[g_{k}^{m}\right]}\\ Y^{\left[g_{k}^{o}\right]} \end{array}\right)\right],$$

where $Y^{\left[g_{j}^{m}\right]}$ is an $m_{j}\times g_{j}$ matrix of missing measurements at the $g_{j}$ gauged sites for the $m_{j}$ time points and $Y^{\left[g_{j}^{o}\right]}$ is an $\left(n-m_{j}\right)\times g_{j}$ matrix of observed measurements at the $g_{j}$ gauged sites for the $\left(n-m_{j}\right)$ time points.

We assume that the response matrix *Y* follows the Gaussian and generalized inverted Wishart model specified by

(1) $$\begin{array}{c} \left\{\begin{array}{ccc} Y|B,\Sigma & ∼ & N(XB,I_{n}⊗\Sigma ),\\ B|\Sigma ,B_{0} & ∼ & N(B_{0},V_{B}⊗\Sigma ),\\ \Sigma & ∼ & GIW(\Theta ,\delta ). \end{array}\right. \end{array}$$

where *B* is an l×(g+u) coefficient matrix with the hyperparameter mean matrix $B_{0}$ and the variance components $V_{B}$, *X* is the matrix of covariates which is defined in (4) and $\left\{\Theta ,\delta \right\}$ is a set of model parameters specified below.

We partition *B* corresponding to the *l* time-varying covariates in conformance with the block structure as

$$B=(B^{\left[u\right]},B^{\left[g_{1}\right]},\cdots ,B^{\left[g_{k}\right]}).$$

By assuming an exchangeable structure across sites, *B* can be written as $B=\tilde{B}R$, where $\tilde{B}$ is the l×d hyperparameter matrix and $R={\left(r_{i,j}\right)}_{d\times (u+g)}$ with $r_{i,j}=1$ for Station *j* under Class *i* and $r_{ij}=0$ otherwise.

Likewise, we partition the (u+g)×(u+g) covariance matrix Σ over gauged and ungauged sites conformably as

$$\begin{array}{c} \Sigma =\left(\begin{array}{cc} \Sigma^{\left[u,u\right]} & \Sigma^{\left[u,g\right]}\\ \Sigma^{\left[g,u\right]} & \Sigma^{\left[g,g\right]} \end{array}\right), \end{array}$$

where $\Sigma^{\left[u,u\right]}$ is a u×u matrix being for the ungauged sites. Further, we partition the g×g covariance matrix $\Sigma^{\left[g,g\right]}$ for the gauged site blocks as follows:

$$\begin{array}{c} \Sigma^{\left[g,g\right]}=\left(\begin{array}{ccc} \Sigma^{\left[g_{1},g_{1}\right]} & \cdots & \Sigma^{\left[g_{1},g_{k}\right]}\\ \vdots & \vdots & \vdots \\ \Sigma^{\left[g_{k},g_{1}\right]} & \cdots & \Sigma^{\left[g_{k},g_{k}\right]} \end{array}\right). \end{array}$$

Similarly, for j=1,⋯,k, we put

$$\begin{array}{c} \Sigma^{\left[g_{j},\cdots ,g_{k}\right]}=\left(\begin{array}{ccc} \Sigma^{\left[g_{j},g_{j}\right]} & \cdots & \Sigma^{\left[g_{j},g_{k}\right]}\\ \vdots & \vdots & \vdots \\ \Sigma^{\left[g_{k},g_{j}\right]} & \cdots & \Sigma^{\left[g_{k},g_{k}\right]} \end{array}\right). \end{array}$$

We reparametrize the matrix Σ through the recursive one-to-one Bartlett transformation for the two blocks:

$$\begin{array}{c} \Sigma =\left(\begin{array}{cc} \Gamma^{\left[u\right]}+{\left(\Upsilon^{\left[u\right]}\right)}^{⊤}\Sigma^{\left[g,g\right]}\Upsilon^{\left[u\right]} & {\left(\Upsilon^{\left[u\right]}\right)}^{⊤}\Sigma^{\left[g,g\right]}\\ \Sigma^{\left[g,g\right]}\Upsilon^{\left[u\right]} & \Sigma^{\left[g,g\right]} \end{array}\right), \end{array}$$

where $\Gamma^{\left[u\right]}=\Sigma^{\left[u,u\right]}-\Sigma^{\left[u,g\right]}{\left(\Sigma^{\left[g,g\right]}\right)}^{-1}\Sigma^{\left[g,u\right]}$ and $\Upsilon^{\left[u\right]}={\left(\Sigma^{\left[g,g\right]}\right)}^{-1}\Sigma^{\left[u,g\right]}$. Similarly, by applying the Bartlett decomposition, we can represent the submatrix $\Sigma^{\left[g_{j},\cdots ,g_{k}\right]}$, for j=1,⋯,k−1, as

$$\begin{array}{c} \Sigma^{\left[g_{j},\cdots ,g_{k}\right]}=\left(\begin{array}{cc} \Gamma_{j}+\Upsilon_{j}^{⊤}\Sigma^{\left[g_{j+1},\cdots ,g_{k}\right]}\Upsilon_{j} & \Upsilon_{j}^{⊤}\Sigma^{\left[g_{j+1},\cdots ,g_{k}\right]}\\ \Sigma^{\left[g_{j+1},\cdots ,g_{k}\right]}\Upsilon_{j} & \Sigma^{\left[g_{j+1},\cdots ,g_{k}\right]} \end{array}\right), \end{array}$$

where $\Gamma_{k}=\Sigma^{\left[g_{k},g_{k}\right]}$ and for j=1,⋯,k−1,

$$\Gamma_{j}:g_{j}\times g_{j}=\Sigma^{\left[g_{j},g_{j}\right]}-\Sigma^{\left[g_{j},\left(g_{j+1},\cdots ,g_{k}\right)\right]}{\left(\Sigma^{\left[g_{j+1},\cdots ,g_{k}\right]}\right)}^{-1}\Sigma^{\left[\left(g_{j+1},\cdots ,g_{k}\right),g_{j}\right]},$$

$$\Upsilon_{j}:\left(g_{j+1}+\cdots +g_{k}\right)\times g_{j}={\left(\Sigma^{\left[g_{j+1},\cdots ,g_{k}\right]}\right)}^{-1}\Sigma^{\left[\left(g_{j+1},\cdots ,g_{k}\right),g_{j}\right]},$$

with

$$\begin{array}{c} \Sigma^{\left[g_{j+1},\cdots ,g_{k}\right]}=\left(\begin{array}{c} \Sigma^{\left[g_{j+1},g_{j}\right]}\\ \vdots \\ \Sigma^{\left[g_{k},g_{j}\right]} \end{array}\right). \end{array}$$

Therefore, the GIW prior distribution for Σ in (1) is equivalently defined in terms of $\left(\Gamma^{\left[u\right]},\Upsilon^{\left[u\right]}\right)$ and $\{\left(\Gamma_{1},\Upsilon_{1}\right),\cdots$, $\left(\Gamma_{k},\Upsilon_{k}\right),\Sigma_{k}\}$ as follows:

(2) $$\begin{array}{c} \left\{\begin{array}{ccc} \Upsilon^{\left[u\right]}|\Gamma^{\left[u\right]} & ∼ & N(\Upsilon_{00},H_{0}⊗\Gamma^{\left[u\right]}),\\ \Gamma^{\left[u\right]} & ∼ & IW(\Lambda_{0},\delta_{0}),\\ \Upsilon_{j}|\Gamma_{j} & ∼ & N(\Upsilon_{0j},H_{j}⊗\Gamma_{j}),\;j=\cdots ,k-1,\\ \Gamma_{j} & ∼ & IW(\Lambda_{j},\delta_{j}),\;j=1,\cdots ,k, \end{array}\right. \end{array}$$

where $\Upsilon^{\left[u\right]}$ is the slope of the optimal linear predictor of $Y^{\left[u\right]}$ based on $Y^{\left[g\right]}$ and $\Gamma^{\left[u\right]}$ is the residual covariance of the optimal linear predictor. Similar interpretations can be applied to $\Upsilon_{j}$ and $\Gamma_{j}$, for j=1,⋯,k−1.

Let H be the set of the hyperparameters in (1) and (2), i.e., $H=\{\Theta ,\delta ,V_{B},B_{0}\}$, where $\Theta =\{\left(\Upsilon_{00},H_{0},\Lambda_{0}\right),\;\left(\Upsilon_{01},H_{1},\Lambda_{1}\right),\cdots ,\left(\Upsilon_{0k-1},H_{k-1},\Lambda_{k-1}\right)$, $\Lambda_{k}\}$ with degrees of freedom parameters $\delta ={\left(\delta_{0},\delta_{1},\cdots ,\delta_{k}\right)}^{⊤}$. Write $H=[H_{u},H_{g}]$. Here, $H_{g}=\{V_{B},B_{0},\left(\Upsilon_{01},H_{1},\Lambda_{1},\delta_{1}\right),$ $\cdots ,\left(\Upsilon_{0,k-1},H_{k-1},\Lambda_{k-1},\delta_{k-1}\right),\left(\Lambda_{k},\delta_{k}\right)\}$, which represents the hyperparameters involved in the marginal distribution of $Y^{\left[g^{o}\right]}$.

If a data matrix appears to be an ascending staircase, the HBST modeling procedure is given as follows:

Step 1. Compute the hyperparameter values that maximize the marginal distribution $f(Y^{\left[g^{o}\right]}|H_{g})$ using an empirical Bayesian approach (see (b) of Appendix A). The EM algorithm is used to obtain ${\hat{H}}_{g}$.
Step 2. Obtain the predictive distributions $f(Y^{\left[g_{k}^{m}\right]}|Y^{\left[g^{o}\right]},{\hat{H}}_{g})$ of missing measurements as in (c) of Appendix A. Fill in the missing data by using the predictive distributions.
Step 3. Obtain the estimate ${\hat{\Sigma }}^{\left[g,g\right]}$ from the estimate of ${\hat{H}}_{g}$. In terms of ${\hat{\Sigma }}^{\left[g,g\right]}$, obtain the estimate of the covariance matrix by using a dimension expansion method given in Qin et al. [5] and the thin-plate spline method given in Wabba and Wendelberger (1980). The details are given in Section 4.1.
Step 4. Estimate the hyperparameters $H_{u}$ and obtain the conditional predictive distribution $f(Y^{\left[u\right]}|Y^{\left[g\right]},\hat{H})$ (see Section 4.2).

## 3. Ozone Concentrations from the Monitoring Stations in Pittsburgh Region

**Figure 1:**
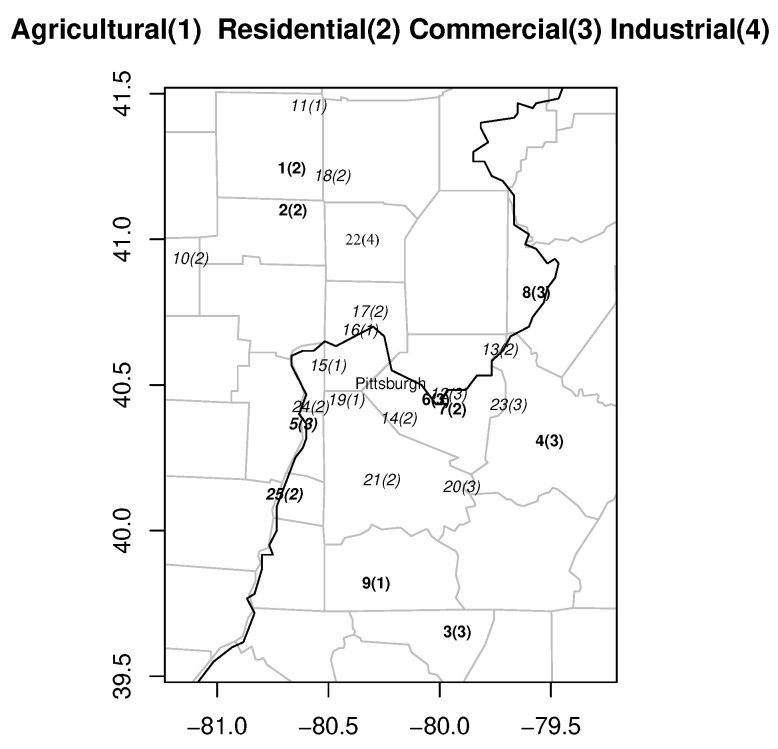
Monitoring stations in the Pittsburgh region.

The ozone concentrations were recorded within $-79^{\circ }$ to $-81.5^{\circ }$ longitude and $39.5^{\circ }$ to $41.5^{\circ }$ latitude around the Pittsburgh region ($-79.23^{\circ }$, $43.39^{\circ }$) for four consecutive summer months, June, July, August and September, over the period from 1995 to 2007. There were 25 monitoring stations in the region as shown in Figure 1, which is the same as Figure 1 in [3]. The original data set $Y_{0}$ was collected from 25 stations, and there were a total of 1586 (13 years × 122 days) measurements at each station. The number of missing data in $Y_{0}$ is shown by N1.Miss in Table 1, which is the same as Table 1 in [3]. In this section, we fill in missing measurements.

### 3.1. Filling in the Missing Measurements for Each Monitoring Station within the Period of Monitoring Blocks

Since there are missing data in the dataset, we follow the steps in [3] in filling in some missing measurements occurred during the operation of each monitoring station, using the regression model as

(3) $$\begin{array}{ccc} \;y_{122(i-1)+j} & = & a\sin\left(\frac{2(122(i-1)+j)\pi }{122}\right)+b\cos\left(\frac{2(122(i-1)+j)\pi }{122}\right)+c_{i}+\varepsilon_{122(i-1)+j}\\ \; & = & a\sin\left(\frac{j\pi }{61}\right)+b\cos\left(\frac{j\pi }{61}\right)+c_{i}+\varepsilon_{122(i-1)+j}, \end{array}$$

for i=1,…,13, and j=1,…,122, where *a* and *b* are regression coefficients, $c_{i}$, for i=1,…,13, are the categorical factors and $\left\{\varepsilon_{t}\right\}$ is a sequence of independently and identically distributed Gaussian random variables with mean 0 and variance $\sigma^{2}$. The model (3) assigns different means to the years with a yearly cycle of 122 days. We re-express the 13 factors in the model via Helmert contrasts, which compare the first level of the factor with all later levels, the second level with all later levels, and so forth. The Helmert matrix, $Z_{13\times 13}$, is defined as follows.

$$\begin{array}{c} Z=\left(\begin{array}{cccccc} 1 & -1 & -1 & \cdots & -1 & -1\\ 1 & 1 & -1 & \cdots & -1 & -1\\ 1 & 0 & 2 & \cdots & -1 & -1\\ \vdots & \vdots & \vdots & \ddots & \vdots & \vdots \\ 1 & 0 & 0 & \cdots & 11 & -1\\ 1 & 0 & 0 & \cdots & 0 & 12 \end{array}\right). \end{array}$$

Let *X*, the matrix of covariates, be

(4) $$\begin{array}{c} X={\left(\begin{array}{cc} S & Z⊗1_{122} \end{array}\right)}_{1586\times 15}, \end{array}$$

where $1_{n}={\left(1,1,\ldots ,1,1\right)}_{1\times n}^{⊤}$ and

$$\begin{array}{c} S={\left(\begin{array}{ccccc} \sin(\pi /61) & \cdots & \sin(i\pi /61) & \cdots & \sin(1586\pi /61)\\ \cos(\pi /61) & \cdots & \cos(i\pi /61) & \cdots & \cos(1586\pi /61) \end{array}\right)}_{2\times 1586}^{⊤}, \end{array}$$

and let $y={\left(y_{1},y_{2},\ldots ,y_{1586}\right)}^{⊤},\;\beta ={\left(a,b,d_{1},\ldots ,d_{13}\right)}^{⊤}$ and $\varepsilon ={\left(\varepsilon_{1},\varepsilon_{2},\ldots ,\varepsilon_{1586}\right)}^{⊤}$ denote the response variables, regression coefficient vector and error variables, respectively. The model (3) is written as y=Xβ+ε.

We then fill in the missing measurements within the blocks by the least squares predictions plus errors and obtain a new data set $Y_{1}$ in which the unfilled missing measurements are either in the end of the time period or in the beginning of the time period. The number of missing data in $Y_{1}$ is shown in Table 1 by N2.Miss.

### 3.2. Filling in the Missing Measurements in $Y_{1}$

To fill in the missing measurements in $Y_{1}$, we can proceed as follows [3]:

(i) Obtain a new data set $Y_{2}$ from $Y_{1}$ by filling in the 488 missing measurements at Stations 5 and 25 during the end of the time period by using the HBST modeling technique. N3.Miss in Table 1 displays the number of missing data in the data set $Y_{2}$, which shows that $Y_{2}$ has a staircase data structure, as all of the missing data are located in the beginning of the time period.
(ii) Put d=4, l=15, n=1586, k=7, $m_{1}=854$, $m_{2}=610$, $m_{3}=488$, $m_{4}=366$, $m_{5}=318$, $m_{6}=244$, $m_{7}=0$, $g_{1}=1$, $g_{2}=1$, $g_{3}=0$, $g_{4}=3$, $g_{5}=1$, $g_{6}=1$ and $g_{7}=16$. Fill in the remaining missing values in $Y_{2}$ by executing Steps 1–2 of the HBST modeling procedure.

## 4. Model the Ozone Concentrations in the Pittsburgh Region

To model ozone concentrations in the Pittsburgh region by spatial interpolation, we cover the region by the 100 grid boxes of a spatial resolution of latitude $0.2^{\circ }\times$ longitude $0.2^{\circ }$. Thus, u=100. The grid points are ungauged sites, and their classes are displayed in Figure 4. To derive the predictive distributions for these grid points, a key step is to estimate the covariance matrix.

### 4.1. Estimation of the Covariance Matrix

In this subsection, we introduce a method for estimating the covariance matrix through a dimension expansion method for modeling the semivariograms in nonstationary fields in terms of ${\hat{H}}_{g}$ from Step 1 of the HBST procedure.

Let $\left\{Y(x)\;:\;x\in S\right\},\;S\in R^{d}$, be an environmental random process, where x is a *d*-dimensional spatial index that varies continuously throughout the region S. At *n* spatial locations denoted by $\left\{x_{i}:i=1,\ldots ,n\right\}$, we observe realizations of the random process Y(x), i.e., $\left\{Y(x_{i}):i=1,\ldots ,n\right\}$. We are interested in learning the spatial dependency of the process through the observed data. The semivariogram function which describes the degree of spatial dependency of an intrinsic stationary random process is a cornerstone in spatial statistics. An intrinsic stationary random process satisfies the following two conditions (Cressie [6]):

1. $E\left(Y(x)\right)=U$, forx∈S,
2. $\mathrm{var}\left(Y\left(x_{i}\right)-Y\left(x_{j}\right)\right)=2\gamma \left(x_{i}-x_{j}\right)$,

where a semivariogram is defined as $\gamma \left(x_{i}-x_{j}\right)=\frac{1}{2}\mathrm{var}\left(Y\left(x_{i}\right)-Y\left(x_{j}\right)\right)$ for two different locations, $x_{i}$ and $x_{j}$, in the monitored region. The estimated covariance matrix of the monitoring stations ${\hat{\Sigma }}^{\left[g,g\right]}$ is based on the estimation of $H_{g}$ from Step 1 of the HBST procedure. We estimate the semivariograms of the ozone concentrations from the monitoring stations by

(5) $$\hat{\gamma }\left(x_{i}-x_{j}\right)=\frac{1}{2}\hat{\mathrm{var}}\left(Y\left(x_{i}\right)\right)+\frac{1}{2}\hat{\mathrm{var}}\left(Y\left(x_{j}\right)\right)-\hat{\mathrm{cov}}\left(Y\left(x_{i}\right),Y\left(x_{j}\right)\right).$$

**Figure 2:**
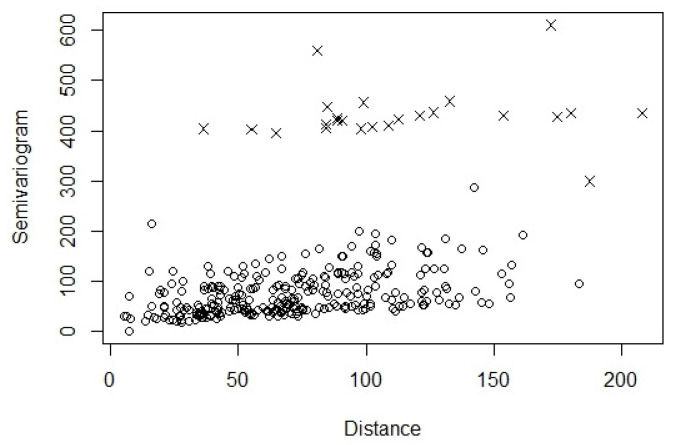
Empirical semivariograms of the ozone concentrations from the monitoring stations versus the Euclidean distances between monitoring stations based on the Bayesian hierarchical model. The semivariograms related to Station 3 are marked by “×”.

From Figure 2, we notice that the estimated semivariograms related to Station 3 (marked by “×”) are much higher than the other stations. We examine the location of Station 3 and notice that it was on the edge of the monitored region. Moreover, there were over ten airports around this station. According to Xue et al. [7], there is a great impact of high-altitude aircrafts on the ozone layer in the stratosphere. This becomes an influential factor in modeling the ozone concentrations. Next, we introduce how this factor is considered in the proposed modeling technique.

It is obvious that this field is not stationary. Bornn et al. [8] proposed a novel approach to find the latent dimensions over which the nonstationary fields exhibit stationarity through dimension expansion. They justified that for a nonstationary Gaussian process Y(x), where $x\in R^{d}$, there exists a vector $z\in R^{p}$, p>0, such that the expanded process $Y\left([x,z]\right)$ is stationary under appropriate moment constraints. Note that [x,z] is the concatenation of the vectors x and z. The stationary semivariogram with latent vectors can be expressed by

$$2\gamma \left(\left[x_{i},z_{i}\right]-\left[x_{j},z_{j}\right]\right)=E{\left(Y\left(\left[x_{i},z_{i}\right]\right)-Y\left(\left[x_{j},z_{j}\right]\right)\right)}^{2},$$

where $\left[x_{i},z_{i}\right]$ is the expanded spatial index for the *i*th location. Qin et al. [5] improved the method in Bornn et al. [8] by considering the covariance structure of the ${\hat{\gamma }}_{i,j},\;\mathrm{for}\;j\neq i$, which are generally correlated. In our application, we use the lasso-penalized weighted least-squares criterion (WLS) in Qin et al. [5] as follows,

(6) $$\begin{array}{cc} {\left(\hat{\phi },Z\right)}_{WLS}= & \underset{Œ,Z}{\mathrm{argmin}}\sum_{j<i}\frac{1}{\gamma_{\phi }^{2}\left(d_{i,j}\left([X,Z]\right)\right)}\left\{{\hat{\gamma }}_{i,j}-\gamma_{\phi }\left(d_{i,j}\left([X,Z]\right)\right)\right\}{}^{2}+\lambda \sum_{k=1}^{p}{∥Z_{.k}∥}_{1}. \end{array}$$

to estimate the parameters and the expanded dimensions. Here, ${\hat{\gamma }}_{i,j}$ is the estimated semivariogram by (5) and $d_{i,j}\left([X,Z]\right)$ is the Euclidean distance between the locations $\left[x_{i},z_{i}\right]$ and $\left[x_{j},z_{j}\right]$ and $Z_{.k}$ is the *k*th column of Z. [X,Z] is the concatenation of the matrices X and Z. The tuning parameter λ in the group lasso is used to determine the number of latent dimensions and regularize the estimation of Z to prevent overfitting. $\gamma_{\phi }\left(d_{i,j}\left([X,Z]\right)\right)$ is a parametric stationary semivariogram model with parameter ϕ. The most popular ones are the exponential model, the spherical model and the Gaussian model (see Journel and Huijbregts [9] and Cressie [6]), among others). For example, the exponential model is defined as

$$\gamma_{\phi }\left(d\right)=\phi_{1}\left(1-\exp\left(-d/\phi_{2}\right)\right)+\phi_{3},$$

where $\phi =\left(\phi_{1},\phi_{2},\phi_{3}\right){}^{⊤},\;\phi_{1}\geq 0,\;\phi_{2}\geq 0$ and $\phi_{3}\geq 0$.

**Figure 3:**
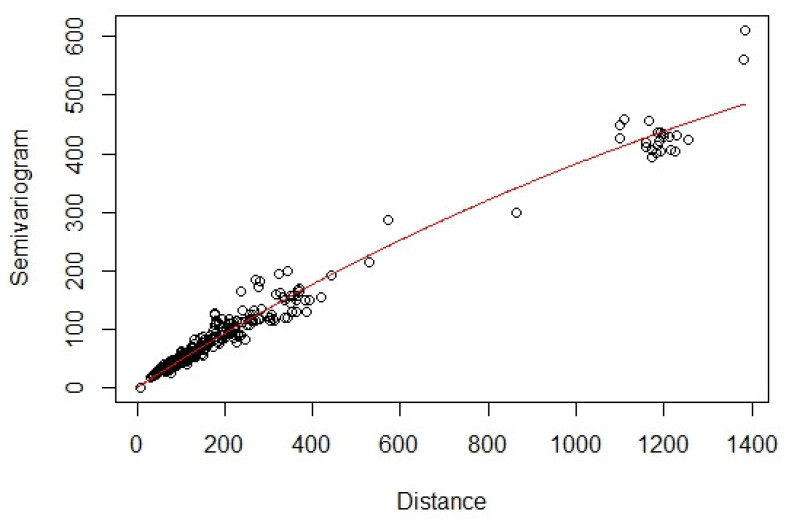
Semivariogram plot of the ozone concentrations from the monitoring stations over a larger range of distances than the range shown in Figure 2, owing to the application of dimension expansion. The fitted exponential semivariogram model is shown by the red solid line.

The semivariogram plot with estimated expanded dimensions (Figure 3) of the monitoring stations shows that the field is in good agreement with the theoretical model, as most of the points are near the solid red line, the fitted exponential semivariogram model. Two extra dimensions are added to the original coordinate with λ=0.01. Figure 3 shows that with the extra dimensions, Station 3 is pushed much further out of the two-dimensional plane, reflecting the impact of high-altitude aircrafts on the ozone layer in the stratosphere we have mentioned earlier.

After the expanded dimensions for the monitoring stations are obtained, we use the thin-plate spline method [10] to estimate the hidden dimensions for the ungauged sites. The semivariograms for the ungauged stations are estimated by the exponential model using the estimated parameter vector $\hat{\phi }$. Next, we estimate the semivariograms $\gamma_{s_{i},s_{j}}$ between stations $s_{i}$ and $s_{j}$ using the exponential model based on the distances over the space composed by the original and the expanded dimensions. Last, the covariance between any two sites can be estimated by

$${\hat{\Sigma }}_{i,j}=\hat{\mathrm{Cov}}\left(Y\left(s_{i}\right),Y\left(s_{j}\right)\right)=\frac{1}{2}{\hat{\sigma }}_{Y(s_{i})}+\frac{1}{2}{\hat{\sigma }}_{Y(s_{j})}-{\hat{\gamma }}_{s_{i},s_{j}},$$

where ${\hat{\sigma }}_{Y(s_{i})}$ and ${\hat{\sigma }}_{Y(s_{j})}$ are estimates of $\sigma_{Y(s_{i})}$ and $\sigma_{Y(s_{j})}$ obtained by the thin-plate spline approach.

### 4.2. Prediction of the Daily Ozone Concentrations at the Grid Points

By Chapter 10 of Le and Zidek (2006), spatial predictive distributions at the grid points given the monitoring sites are as follows:

(7) $$\begin{array}{c} \left(Y^{\left[u\right]}|Y^{\left[g\right]},H\right)∼t_{n\times u}\left(U^{u|g},\frac{\Phi^{\left[u|g\right]}⊗\Psi^{\left[u|g\right]}}{\delta_{0}^{*}},\delta_{0}^{*}\right), \end{array}$$

where $\delta_{0}^{*}=\delta_{0}-u+1$, $\Psi^{\left[u|g\right]}=\Lambda_{0}$, $U^{\left[u|g\right]}=XB_{0}^{\left[u\right]}+\left(Y^{\left[g\right]}-XB_{0}^{\left[g\right]}\right)\Upsilon_{00}$ and $\Phi^{\left[u|g\right]}=I_{n}+XV_{B}X^{⊤}+\left(Y^{\left[g\right]}-XB_{0}^{\left[g\right]}\right)H_{0}{\left(Y^{\left[g\right]}-XB_{0}^{\left[g\right]}\right)}^{⊤}$ (see (a) of Appendix A for definition of the matric-*t* distribution).

We estimate the hyperparameters associated with the grid points $\Lambda_{0},\;\Upsilon_{00},\;H_{0}$ and $\delta_{0}$ via

$${\hat{\delta }}_{0}=\frac{{\hat{\delta }}_{1}+\cdots +{\hat{\delta }}_{k}}{k},\;{\hat{H}}_{0}={\hat{\Lambda }}^{\left[1,\cdots ,k\right]},\;{\hat{\Upsilon }}_{00}={\left({\hat{\Sigma }}^{\left[g,g\right]}\right)}^{-1}{\hat{\Sigma }}^{\left[g,u\right]},$$

$${\hat{\Lambda }}_{0}=\frac{{\hat{\delta }}_{0}-u-1}{1+tr({\hat{\Sigma }}^{\left[g,g\right]}{\hat{H}}_{0})}\left({\hat{\Sigma }}^{\left[u,u\right]}-{\hat{\Upsilon }}_{00}^{⊤}{\hat{\Sigma }}^{\left[g,g\right]}{\hat{\Upsilon }}_{00}\right)$$

with

$$\begin{array}{ccc} {\hat{\Lambda }}^{\left[j,\cdots ,k\right]}=\left(\begin{array}{cc} {\hat{\Lambda }}_{j}+{\hat{\Upsilon }}_{0j}^{⊤}{\hat{\Lambda }}^{\left[j+1,\cdots ,k\right]}{\hat{\Upsilon }}_{0j} & {\hat{\Upsilon }}_{0j}^{⊤}{\hat{\Lambda }}^{\left[j+1,\cdots ,k\right]}\\ {\hat{\Lambda }}^{\left[j+1,\cdots ,k\right]}{\hat{\Upsilon }}_{0j} & {\hat{\Lambda }}^{\left[j+1,\cdots ,k\right]} \end{array}\right) & , & \;j=1,\ldots ,k-1, \end{array}$$

and ${\hat{\Lambda }}^{\left[k\right]}={\hat{\Lambda }}_{k}$.

After all of the hyperparameters in the predictive distributions are estimated, we can predict the daily ozone concentrations at all the grid points in the time period of study by generating samples from the predictive distributions.

## 5. Environmental Network Extension

Assume that *Y* has the density function *f*. The total reduction in uncertainty of *Y* can be presented by the entropy of its distribution, i.e., H(Y)=−E[logf(Y)/h(Y)], where h(·) is a not necessarily integrable reference density (Jaynes [11]). According to the predictive distribution (7), the total entropy $H(Y^{\left[u\right]}|Y^{\left[g\right]})$ can be defined as

(8) $$\begin{array}{c} H\left(Y^{\left[u\right]}|Y^{\left[g\right]}\right)=\frac{1}{2}\log\left(\left|\Psi^{\left[u|g\right]}\right|\right)+c_{u}\left(u,q\right), \end{array}$$

where $c_{u}\left(u,q\right)$ is a constant depending on the degree of freedom and the dimension of the ungauged sites.

The key step in expanding an environmental network is to find appropriate ungauged sites to add to the existing network that maximizes the corresponding entropy. We use the following optimality criterion as given in [3]:

(9) $$\begin{array}{c} \max_{add}{\left(\frac{1}{2}\log\left|\Psi^{\left[u|g\right]}\right|\right)}^{add} \end{array}$$

**Figure 4:**
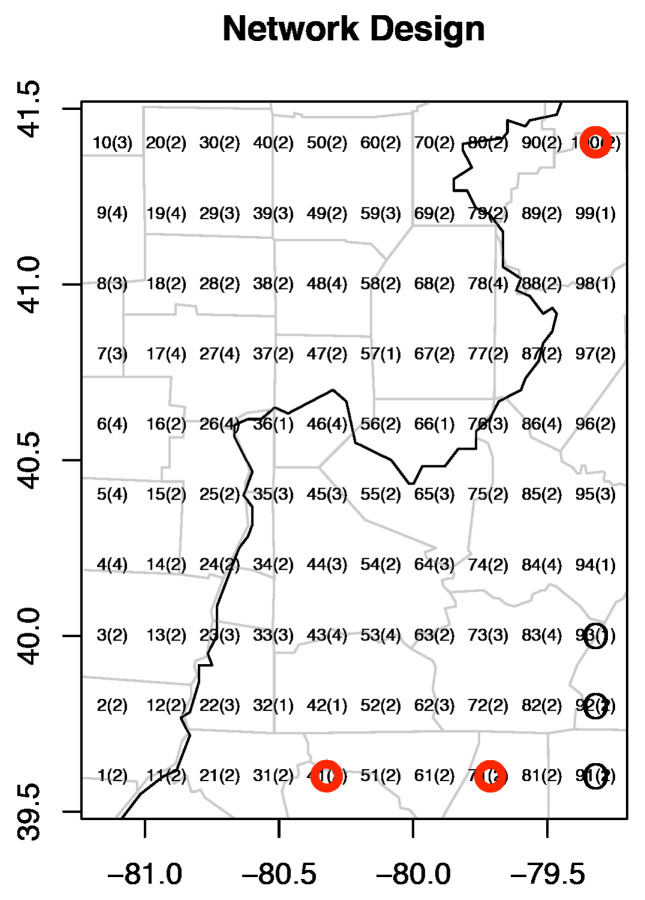
The selected sites among 100 grid points (black circled points by [3] and red circled points by our method).

The add sites, in a vector of dimension $u_{1}$, are selected to maximize the entropy in (8). In [3], the grid points $\left\{91,92,93\right\}$ were selected with the highest entropy 11.3774. The proposed method selects the grid points $\left\{41,71,100\right\}$ with entropy 12.1207. This selection is more reasonable, as they are not gathered in the southeast corner of the region like $\left\{91,92,93\right\}.$ The selected sites among 100 grid points by the two methods are shown in Figure 4 below.

## 6. Model Evaluation

In this section, we use the leave-one-out cross-validation to evaluate the accuracy of the predictive model derived using the proposed method and compare the proposed method with the one in [3]. We select the observations from one of the original 25 stations as validation data, and observations in the remaining 24 stations are treated as training data. We use the data from day 855 to day 1586 at the end of the study from each station to evaluate the prediction because during this period, none of the stations has missing data. By choosing this period, we avoid using the Bayesian hierarchical modeling technique for estimating the missing data in the training data set, which is time-consuming and not our intention for evaluating the proposed method on estimating the covariance matrix. Station 22 is excluded because it is the only industrial station in the study. For each of the 24 stations, we generate 100 samples from the predictive distribution with parameters estimated using observations from the rest of the 23 stations. We compute the average of relative absolute bias (ARAB) as $\sum_{j=1}^{100}\left|\left(y_{j,i,t}-y_{i,t}\right)/y_{i,t}\right|$, where $y_{j,i,t}$ is the *j*th sample generated from the predictive distributions and $y_{i,t}$ is the observation from Station *i* on time *t*. The results are given in Table 2.

In Table 2, “-” means that there is no prediction for the station because there are negative correlations and the method in [3] is not applicable to estimate the predictive distribution. The results in Table 2 show that the proposed method provides slightly more accurate predictions than the one in [3] for most of the stations. More important is that, when there are negative correlations obtained from the estimations of the hierarchical Bayesian spatio-temporal modeling technique, the method in [3] fails to estimate the covariance matrix, while the proposed method still provides accurate predictions except for Station 3. This is expected because Station 3 is an influential station. Therefore, if we use observations at Station 3 as the validation data set, it has a great impact on estimating the covariance matrix.

## 7. Conclusions

In this paper, we have derived a predictive model through the hierarchical Bayesian spatio-temporal modeling technique given in [12] at ungauged sites based on the covariance matrix estimated by a dimension expansion method for modeling semivariograms in nonstationary fields. Further, we have applied an entropy criterion (see [12] or [3] for details) based on the predictive model to decide if new stations need to be added. This entropy criterion helps to solve the environmental network design problem. For demonstration, we have applied the proposed method on ozone concentrations at 25 stations in the Pittsburgh region studied in [3]. The proposed method has provided satisfactory results. Moreover, the results have shown that the method is more general and applicable, as no assumption is imposed on the correlation structure.

## Figures and Tables

**Table 1:** Location of the stations and number of missing data.

ID	Class	Lon	Lat	N1.Miss	N2.Miss	N3.Miss	ID	Class	Lon	Lat	N1.Miss	N2.Miss	N3.Miss
1	2	−40.24	80.66	855	854	854	14	2	−40.38	80.18	22	0	0
2	2	−41.09	80.65	610	610	610	15	1	−40.56	80.50	13	0	0
3	3	−39.64	79.92	618	610	610	16	1	−40.68	80.35	11	0	0
4	3	−40.30	79.50	488	488	488	17	2	−40.74	80.31	4	0	0
5	3	−40.36	80.61	858	854	366	18	2	−41.21	80.48	5	0	0
6	3	−40.44	80.01	370	366	366	19	1	−40.44	80.42	16	0	0
7	2	−40.41	79.94	370	366	366	20	3	−40.14	79.90	3	0	0
8	3	−40.81	79.56	328	318	318	21	2	−40.17	80.26	1	0	0
9	1	−39.81	80.28	278	244	244	22	4	−40.99	80.34	0	0	0
10	2	−40.93	81.12	12	0	0	23	3	−40.42	79.69	5	0	0
11	1	−41.45	80.59	1	0	0	24	2	−40.42	80.58	5	0	0
12	3	−40.46	79.96	2	0	0	25	2	−40.12	80.69	488	488	0
13	2	−40.61	79.73	8	0	0							

The numbers 1, 2, 3 and 4 under Class denote agricultural, residential, commercial and industrial, respectively.

**Table 2:** Mean and SD of the average of relative absolute bias.

ID	Our Method	Jin et al. (2012) [3]	ID	Our Method	Jin et al. (2012) [3]
1	0.0789 (0.0627)	0.8134 (0.0682)	13	0.1145 (0.1096 )	0.2003 (0.1769)
2	0.1206 (0.1356)	0.1221 (0.1121)	14	0.1361 (0.1732)	0.2211 ( 0.2283)
3	0.8517 (0.8517)	0.1572 ( 0.1572)	15	0.1911 (0.2052)	-
4	0.1756 (0.1693)	-	16	0.1189 (0.1179)	0.1285 (0.1161)
5	0.1575 (0.1731)	0.1986 (0.1855)	17	0.1496 (0.1594 )	0.1669 (0.1727)
6	0.1336 (0.1513)	0.1477 (0.1667 )	18	0.1253 (0.1154 )	0.1256 (0.1372)
7	0.1265 (0.1563 )	0.1456 (0.1732)	19	0.1369 (0.1272)	0.1026 ( 0.0994)
8	0.0968 (0.0804)	0.1135 (0.1023)	20	0.1603 (0.1598)	0.1310 (0.1134)
9	0.1497 (0.1104)	0.1619 (0.1208)	21	0.1351 (0.1154)	0.1274 (0.1123)
10	0.1589 (0.1796 )	-	23	0.1617 (0.1858)	-
11	0.6913 (0.6455)	-	24	0.1286 (0.1051)	-
12	0.1406 (0.1409)	0.1265( 0.1416)	25	0.1583 (0.1701)	0.1722 ( 0.1675)

## Data Availability

The data that support the findings of this study are available from the corresponding author, upon reasonable request.

## Appendix A. Hierarchical Bayesian Spatio-Temporal Modeling

The following is mainly based on Chapter 10 of Le and Zidek (2006).

(a) Somedistributions

*Matrix normal distribution*. If, for an n×m matrix U and positive definite matrices A:n×n and B:m×m, the density function of an n×m random matrix *X* has the form

$$\begin{array}{c} f\left(X\right)={\left(2\pi \right)}^{-\frac{nm}{2}}{\left|A\right|}^{-\frac{m}{2}}{\left|B\right|}^{-\frac{n}{2}}\exp\left\{-\frac{1}{2}\mathrm{tr}\left[A^{-1}\left(X-U\right)B^{-1}{\left(X-U\right)}^{⊤}\right]\right\}, \end{array}$$

then *X* is said to have a matrix normal distribution and is denoted by $X∼N_{n\times m}\left(U,A⊗B\right)$.

*Inverted Wishart distribution*. If, for a p×p positive definite matrix Ψ and a positive constant δ≥p, the density function of a p×p random matrix *S* has the form

$$f\left(S\right)=\frac{1}{2^{\frac{\delta p}{2}}\Gamma_{p}\left(\frac{\delta }{2}\right)}{\left|\Psi \right|}^{\frac{\delta }{2}}{\left|S\right|}^{\frac{-(\delta +p+1)}{2}}\exp\left\{-\frac{1}{2}\mathrm{tr}\left(S^{-1}\Psi \right)\right\},$$

then *S* is said to have an inverted Wishart distribution and is denoted by S∼IW(Ψ,δ).

*Matric-t distribution*. If, for an n×m matrix U, positive definite matrices A:n×n and B:m×m and a positive constant δ>0, the density function of an n×m random matrix *X* has the form

$$f\left(X\right)\propto {\left|A\right|}^{-\frac{m}{2}}{\left|B\right|}^{-\frac{n}{2}}{\left|I_{n}+\delta^{-1}A^{-1}\left(X-U\right)B^{-1}{\left(X-U\right)}^{⊤}\right|}^{-\frac{\delta +n+m-1}{2}},$$

then *X* is said to have a matric-*t* distribution and is denoted by $X∼t_{n\times m}\left(U,A⊗B,\delta \right)$.

(b) Estimation of $H_{g}=\left\{V_{B},B_{0},\left(\Upsilon_{01},H_{1},\Lambda_{1},\delta_{1}\right),\cdots ,\left(\Upsilon_{0,k-1},H_{k-1},\Lambda_{k-1},\delta_{k-1}\right),\left(\Lambda_{k},\delta_{k}\right)\right\}$

Le and Zidek (2006) showed that the predictive distributions derived through the integrated framework above are completely characterized by their hyperparameters, which are estimated by an empirical Bayes approach, that is, to estimate them by maximizing the marginal likelihood of all the measured responses (conditional on those hyperparameters) evaluated at their observed values. This procedure is referred to as type-II maximum likelihood estimation (type-II MLE). To estimate $H_{g}$, the following procedure can be employed: Compute the hyperparameter values that maximize the marginal distribution $f(Y^{\left[g^{o}\right]}|H_{g})$, where $Y^{\left[g^{o}\right]}=\left\{Y^{\left[g_{1}^{o}\right]},\cdots ,Y^{\left[g_{k}^{o}\right]}\right\}$. The subscript *g* indicates that not all the hyperparameters are involved in this marginal distribution. The response matrix *Y* follows the GIW distribution specified by (1) and (2). The marginal distribution $f(Y^{\left[g^{o}\right]}|H_{g})$ can be written as

(A1) $$\begin{array}{c} Y^{\left[g^{o}\right]}|H_{g}∼\prod_{j=1}^{k}t_{\left(n-m_{j}\right)\times g_{j}}\left(U_{o}^{\left[j\right]},\Phi_{o}^{\left[j\right]}⊗\Psi_{o}^{\left[j\right]},\delta_{o}^{\left[j\right]}\right), \end{array}$$

where $U_{o}^{\left[j\right]}=U_{\left(2\right)}^{\left[j\right]}$, $\Phi_{o}^{\left[j\right]}=A_{22}^{\left[j\right]}$, $\Psi_{o}^{\left[j\right]}=\frac{\Lambda_{j}}{\delta_{j}-g_{j}+1}$, $\delta_{o}^{\left[j\right]}=\delta_{j}-g_{j}+1$ with ${\tilde{\varepsilon }}^{\left[g_{j+1},\cdots ,g_{k}\right]}=Y^{\left[g_{j+1},\cdots ,g_{k}\right]}-XB_{0}^{\left[g_{j+1},\cdots ,g_{k}\right]}$, for j=1,⋯,k−1,

$$\begin{array}{c} \left(\begin{array}{c} U_{\left(1\right)}^{\left[j\right]}\\ U_{\left(2\right)}^{\left[j\right]} \end{array}\right):\left(\begin{array}{c} m_{j}\times g_{j}\\ \left(n-m_{j}\right)\times g_{j} \end{array}\right)=XB_{0}^{\left[g_{j}\right]}+{\tilde{\varepsilon }}^{\left[g_{j+1},\cdots ,g_{k}\right]}\Upsilon_{0j}, \end{array}$$

and

$$\begin{array}{ccc} \left(\begin{array}{cc} A_{11}^{\left[j\right]} & A_{12}^{\left[j\right]}\\ A_{21}^{\left[j\right]} & A_{22}^{\left[j\right]} \end{array}\right) & : & \left(\begin{array}{cc} m_{j}\times m_{j} & m_{j}\times \left(n-m_{j}\right)\\ \left(n-m_{j}\right)\times m_{j} & \left(n-m_{j}\right)\times \left(n-m_{j}\right) \end{array}\right)\\ & = & I_{n}+XV_{B}X^{⊤}+{\tilde{\varepsilon }}^{\left[g_{j+1},\cdots ,g_{k}\right]}H_{j}{\left({\tilde{\varepsilon }}^{\left[g_{j+1},\cdots ,g_{k}\right]}\right)}^{⊤}. \end{array}$$

Although $f(Y^{\left[g^{o}\right]}|H_{g})$ can be written as a matric-*t* distribution as in (A1), direct maximization of this marginal density presents a challenge. The EM algorithm helps circumvent it.

(c) Predictive distributions of missing data

By Theorem 10.1 of Le and Zidek (2006), it follows that

(A2) $$\begin{array}{c} Y^{\left[g_{k}^{m}\right]}|Y^{\left[g^{o}\right]},H_{g}∼t_{m_{k}\times g_{k}}\left(U_{\left(m|g\right)}^{\left[k\right]},\Phi_{\left(m|g\right)}^{\left[k\right]}⊗\Psi_{\left(m|g\right)}^{\left[k\right]},\delta_{\left(m|g\right)}^{\left[k\right]}\right), \end{array}$$

(A3) $$\begin{array}{c} Y^{\left[g_{j}^{m}\right]}|Y^{\left[g_{j+1}^{m},\cdots ,g_{k}^{m}\right]},Y^{\left[g^{o}\right]},H_{g}∼t_{m_{j}\times g_{j}}\left(U_{\left(m|g\right)}^{\left[j\right]},\Phi_{\left(m|g\right)}^{\left[j\right]}⊗\Psi_{\left(m|g\right)}^{\left[j\right]},\delta_{\left(m|g\right)}^{\left[j\right]}\right), \end{array}$$

where $U_{\left(m|g\right)}^{\left[j\right]}=U_{\left(1\right)}^{\left[j\right]}+A_{12}^{\left[j\right]}{\left(A_{22}^{\left[j\right]}\right)}^{-1}\left(Y^{\left[g_{j}^{o}\right]}-U_{\left(2\right)}^{\left[j\right]}\right)$, $\Phi_{\left(m|g\right)}^{\left[j\right]}=\frac{\delta_{j}-g_{j}+1}{\delta_{j}-g_{j}+n-m_{j}+1}$$\left[A_{11}^{\left[j\right]}-A_{12}^{\left[j\right]}{\left(A_{22}^{\left[j\right]}\right)}^{-1}A_{21}^{\left[j\right]}\right]$, $\Psi_{\left(m|g\right)}^{\left[j\right]}=\frac{1}{\delta_{j}-g_{j}+1}\left[\Lambda_{j}+{\left(Y^{\left[g_{j}^{o}\right]}-U_{\left(2\right)}^{\left[j\right]}\right)}^{⊤}{\left(A_{22}^{\left[j\right]}\right)}^{-1}\left(Y^{\left[g_{j}^{o}\right]}-U_{\left(2\right)}^{\left[j\right]}\right)\right]$ and $\delta_{\left(m|g\right)}^{\left[j\right]}=\delta_{j}-g_{j}+n-m_{j}+1$.
